# Combating weight-stigmatization in online spaces: the impacts of body neutral, body positive, and weight-stigmatizing TikTok content on body image and mood

**DOI:** 10.3389/fpsyt.2025.1577063

**Published:** 2025-06-10

**Authors:** Raeanna Kilby, Kristin D. Mickelson

**Affiliations:** Arizona State University, New College of Interdisciplinary Arts and Sciences, Glendale, AZ, United States

**Keywords:** body positivity, body neutrality, weight-stigma, TikTok, social media, self-objectification, mood, body image

## Abstract

Social media movements centered on body positivity and body neutrality both encourage healthy attitudes toward the physical body; however, these movements are conceptually distinct and may have unique influences on body image. This study examined how brief exposure to different types of body acceptance and weight-stigmatizing content affects body image and mood. Participants consisted of women and gender-diverse individuals (N = 326) who completed an online survey and were randomly assigned to view one of four TikTok video compilations: body neutrality, body positivity, weight-stigmatizing, or travel (control) content. Exposure to body positivity and body neutrality content was associated with improvements in functional appreciation, self-objectification, body dissatisfaction, and negative affect. Moreover, although participants across body-focused conditions reported thinking about their appearance to a similar extent, those in the body-positive and body-neutral groups reported more frequent positive appearance-related thoughts than those in the weight-stigmatizing or control conditions. Importantly, differences emerged between the two body acceptance conditions, such that body neutrality was uniquely effective in reducing self-objectification relative to weight-stigmatizing content, while body positivity significantly enhanced positive affect. Moderation analyses revealed marginal interactions, suggesting that the effect of content on body dissatisfaction varied by gender identity, while positive affect varied by perceived body silhouette. Overall, these findings indicate that body-positive and body-neutral content on TikTok may serve as beneficial alternatives to weight-stigmatizing media, though each approach may yield distinct benefits especially in consideration of individual identity characteristics.

## 1 Introduction

### 1.1 Social media and weight-stigma

Since its emergence in the 1990s, social media has become widespread, with upwards of 72% of all Americans using at least one social media site (1). Despite the high prevalence rate, one-third of Americans believe social media use (SMU) has overall negative impacts on mental health (2). These feelings of apprehension towards social media are not unfounded, with abundant research demonstrating potential detrimental effects of SMU on users’ well-being (see 3, for an extensive review). Of these concerns, one of the most salient is social media’s impact on body image. Initially, researchers assumed that overall time on social media was the sole predictor of body image; however, more recently, research suggests that the content users are exposed to and engaging with may be a much larger predictor than overall time spent on SMU (4–6). Specifically, content that centers around weight loss (7) or idealized beauty standards has been found to be the most detrimental to self-esteem and body image (8–11).

While beauty standards have evolved over time, the thin ideal remains one of the most pervasive and influential in Western societies. The thin ideal is a concept created and perpetuated by social norms and expectations, depicting an ultra-slender and toned female body as the epitome of success, desirability, and happiness (12). While society has long placed importance on thinness, the increasing integration of media into our daily lives has amplified this phenomenon by exposing individuals to a constant, and often inescapable stream of beauty ideals. Indeed, research shows that social media’s influence on body ideals is greater today than traditional broadcast media (13–16). This reflects what Hepp (17) describes in his theory of deep mediatization, in which networked digital media is an active force shaping societal structures, personal identities, and cultural norms. In the context of body ideals, this means that beauty standards are not only disseminated more widely but are also deeply integrated into how individuals perceive femininity and social value within themselves and others through body type, making appearance ideals appear more pervasive and inescapable than ever before.

Idealized body expectations not only influence how individuals view themselves but also how society views and treats those who do not conform. One of the most pervasive consequences of internalized body standards is the stigmatization of individuals with larger bodies. A critical aspect of the thin ideal is that those able to achieve thinness are not only successful, desirable, and happy, but strong-willed and in control of their bodies (18). Conversely, those who fall outside of the ideal weight, especially plus-size women, are stigmatized as lazy and lacking willpower (19). Consequently, the stigmatization of those with larger bodies, also referred to as weight-stigma, has resulted in pervasive and widespread discrimination (20). Concerningly, stigmatization and discrimination against those with large bodies has continued to grow, increasing by 66% nationally from 1995 to 2006 (21), with the common perception being that discrimination is a useful tool to increase “healthier lifestyles,” despite not being supported by research (20).

Stigmatization and discrimination of people with larger bodies is also extremely prevalent within online communities through body shaming comments and weight-bias content (22). The combination of anonymity and lack of repercussions from these platforms lowers individuals’ adherence to social norms, resulting in much more extreme forms of weight-stigma than in non-online spaces. A study by Jeon et al. (23) found that for individuals with larger bodies, body shaming comments were found to be twice as likely than comments defending these individuals. Furthermore, the content existing on social media can be weight-biased in nature, expressing negative attitudes or stereotypes towards larger bodies and idealizing thin bodies. An analysis of posts and comments on major social media sites found that 92% of content relating to larger bodies used the word “fat” and was most often associated with negative connotations (24).

Unsurprisingly, increased consumption and internalization of weight-stigmatizing content through social media use (SMU) have consistently been shown to have a unidirectional association with worse mood and body image concerns across all body types, even during instances of acute exposure (see 4–6 for extensive literature reviews). However, this is partially heighted for individuals with larger bodies, who face immense pressure to critically examine and disparage their own bodies. Consequently, several studies have found that individuals with larger bodies report higher levels of body dissatisfaction (25, 26). More concerning is that this negative body image is a significant risk factor for the development of disordered eating behaviors and related deficits across various domains, including physical health, social relationships, emotional well-being, academic performance, and professional success (27, 28).

### 1.2 TikTok – personalized problematic content

However, not all social media platforms are alike, as users engage with problematic body-related content in different ways across platforms. While much of the research has traditionally centered on Instagram, growing concerns are now being raised about the impact of TikTok (29). As a highly popular, short-form video platform, TikTok has rapidly grown into a mainstream source for appearance and body ideals. With 150 million U.S. users—nearly half the population (30)—and the highest average screen time of any platform at 26 hours per month (31), TikTok wields significant influence, particularly over its predominantly young female user base (32).

Like other image-centric platforms, TikTok is riddled with a constant stream of appearance and body-focused content, contributing to unrelenting beauty trends (33–35). However, what makes TikTok uniquely troubling in comparison to other platforms is its highly algorithmically driven presentation of content. In other words, TikTok utilizes an algorithm based on data taken from user interactions, such as accounts followed, likes, comments, and saved videos to personalize video recommendations (29, 36). This individualized approach means that users—particularly those already vulnerable to body dissatisfaction—are frequently exposed to and encouraged to engage with harmful content. A study by the Center for Countering Digital Hate (37) highlights the extent of this issue, demonstrating that TikTok’s algorithm can rapidly identify and exploit body image-related insecurities. When simulated accounts mimicking 13-year-old girls engaged with body image and mental health content, the algorithm began promoting eating disorder-related videos within just eight minutes. This suggests that TikTok not only promotes body ideals and weight-stigma, but actively shapes and intensifies such beliefs, even encouraging unhealthy behaviors to achieve thinness, contributing to widespread body image concerns and subsequent eating disorder behaviors among its users (38, 39). Hence, given TikTok’s power to influence attitudes and behaviors regarding the body ideal, it is crucial to critically examine its role in promoting body image concerns and to explore potential solutions.

### 1.3 Body positivity and body neutrality on TikTok

Just as TikTok has the power to shape beliefs and behaviors in ways that contribute to weight-stigma, body dissatisfaction, and disordered eating, it also has the potential to foster more positive relationships with the body. While much of the platform’s content reinforces unrealistic body ideals and weight-stigma, an increasing number of users are engaging with content that challenges these standards. This shift is reflected in the rise of body acceptance content, including both body positivity (BoPo) and body neutrality. Although body positivity and body neutrality share the common goal of reducing body image concerns, they differ in their approaches. The central ideology of BoPo is that all individuals, regardless of shape and size, deserve to have a positive relationship with their physical body (40). BoPo promotes self-compassion and self-acceptance by loving and embracing the body including all its perceived flaws (41). For instance, someone practicing BoPo will have beliefs such as “I feel good about myself because I know I am beautiful, flaws and all” or “I love my stomach and its stretch marks.”

Body neutrality, on the other hand, shifting focus away from beauty and prioritizes overall well-being and functionality. Pellizzer and Wade (42) proposed a working definition of body neutrality that is made up of three main components. First, body neutrality encourages individuals to step away from their appearance judgements entirely, in that a person’s body is neither inherently good nor bad. Essentially, it takes away all appearance-based judgments, either positive or negative, from the body. Second, body neutrality encourages individuals to find self-worth in their intrinsic qualities and extrinsic passions, not in their appearance. For instance, someone practicing body neutrality may have beliefs such as “How I feel about myself has nothing to do with my appearance.” Lastly, body neutrality encourages individuals to focus on valuing the functionalities of their bodies, rather than the appearance of their bodies, a practice termed functional appreciation. Importantly, functional appreciation is not limited to those with able-bodies, in that those with physical limitations still have bodies capable of functioning, though that functionality may manifest in different ways (43). Moreover, while functional appreciation includes physical capabilities, it also encompasses other functions, such as the body’s experience and ability to engage with the world (e.g. expression, connection, and communication) (44).

Most research has focused on the effects of body positivity, finding support for body positivity as an alternative to harmful body content. Indeed, experimental studies have found improvements in body image concerns and mood following exposure to body positivity (40, 45–48). However, criticisms have been raised regarding BoPo’s continued focus on appearance. Specially, users have criticized BoPo content, sharing sentiments like, “*I was never insecure about my stretch marks until people shoved it down my throat that they’re beautiful*,” and “*I was never insecure until I was told to love parts of my body that I didn’t think twice about*” (49). As a result, while BoPo aims to foster body acceptance, its emphasis on appearance can inadvertently exacerbate body dissatisfaction for some.

These sentiments are reflected within recent research, showing that even brief exposure to BoPo content may lead to a boomerang effect, increasing upward appearance comparisons and self-objectification (33, 40, 50). Originally proposed by Fredrickson and Roberts (51), objectification is treating a person as an object that can be used and manipulated, as opposed to an individual with agency (52). When objectified, individuals are stripped of their personhood until they exist as just a body to be evaluated by others (53). When individuals internalize body objectification, or *self-objectify*, they become accustomed to viewing themselves as their physical body through the lens of an observer. Individuals who self-objectify may have thoughts such as “My value comes from my appearance” or “I will not be satisfied with myself until I reach the ideal societal body.”

Notably, positive body image, rooted in appreciation, respect, and care for the body, is distinct from self-objectification (54–56). Positive body image, which recognizes a broad understanding of beauty, is internally motivated and self-affirming, whereas self-objectification is externally focused and performative. However, the distinction between self-objectification and positive body image can become blurred—particularly for individuals shifting away from internalized thin-ideal messaging, where their self-worth has long been conditioned to be contingent by others’ evaluations. For these individuals, body-positive content, while well-intentioned, may still reinforce performative relations with the body if the sense of empowerment is contingent on others’ recognizing wider definitions of beauty rather than one’s own internal acceptance.

Furthermore, a core component of positive body image is appreciation of the body beyond its appearance—such as functional appreciation—a nuance that is underemphasized in much of BoPo content (40, 57). Hence, despite BoPo’s central tenets focusing on creating positive relationships with the physical body, its focus on appearance can inadvertently reinforce self-objectification by keeping physical appearance central to one’s self-image and worth (40, 58). Importantly, this does not mean that body positivity is inherently harmful. In fact, for many, it serves as an empowering and affirming counter-narrative to idealized body content. However, for those with more complex or conflicted relationships with their body, including those who have deeply internalized the thin ideal, body positivity may not feel attainable or appropriate. For some, body neutrality may even serve as a steppingstone, providing a non-performative mindset that supports healing and acceptance until a positive, self-affirming relationship with the body, such as demonstrated in BoPo, can be developed. It is therefore crucial for research to explore a range of body acceptance content, such as body neutrality, to address a wider diversity of needs.

There are currently only two studies, to our knowledge, that look at exposure to digital body neutrality content in connection with body satisfaction (59, 60). Both studies involved exposing participants to a single session of body neutral content and demonstrated improvements in functional appreciation and body satisfaction. Improvements in mood as well as fewer upward appearance comparisons were also reported. Importantly, the study performed by Seekis and Lawrence (59) focused on body-neutral TikTok content, providing support for the efficacy of body-neutral content on video-central social media platforms. Notably, its impact on self-objectification, especially in relation to body positivity and traditional body idealizing content, has not been explored.

### 1.4 Body silhouette and gender-diverse populations

Notably, no study has directly compared the effects of body positivity and body neutrality, especially in consideration of unique user identities and needs. By examining how these approaches differ in their impact, researchers can better identify strategies that promote healthier and more inclusive relationships with the body. For instance, for those with more complicated reactions to the body, where the idea of unconditional love is not necessarily practical, body neutrality may be an easier ideology to adopt. This is especially applicable to individuals with chronic illnesses or disabilities, who often report feeling betrayed by their bodies (61), those with or recovering from an eating disorder, or populations at higher risk for body concerns. Research has identified several characteristics that elevate the risk for body image concerns, one of the most prominent being body type (26). Specifically, those with larger bodies often report higher body dissatisfaction, due to the previously mentioned promotion of a thin body ideal and pervasiveness of weight-stigma within general society and online spaces.

Hence, body positivity and body neutrality are important in that they challenge the normality of weight-stigmatization in online spaces. However, the combination of higher body dissatisfaction and experiences of stigmatization among those with larger bodies may differentially impact the effectiveness of body-neutral and body-positive content. Therefore, research is needed to see if body-neutral and body-positive content are equally effective strategies, especially in consideration of body types, before being recommended to users regardless of individual characteristics.

In addition to those with larger bodies, transgender and gender-diverse individuals (TGD) also show elevated rates of body dissatisfaction. Indeed, studies have found that upwards of 70% of transgender and gender diverse participants reported experiencing body dissatisfaction, with transgender youth exhibiting higher levels of eating disorder behaviors and diagnoses compared to their cisgender peers (62). Despite these higher prevalence rates, there is a lack of research on prevention and treatment within TGD communities. Regarding body acceptance movements for TGD communities, body positivity has been heavily critiqued by community advocates and researchers alike for being exclusionary and invalidating regarding body dissatisfaction due to gender dysphoria (63–67). Conversely, there has been preliminary support for body neutrality in relation to body dissatisfaction for TGD communities (68). Smith et al. (60) found that a single-session intervention of exposure to body-neutral content, for individuals experiencing body and mood disturbances, improves body image. Those included in the sample were diverse in their gender identity, including 32% identifying as non-binary and almost 15% identifying as transgender. Preliminary support has also been found within qualitative works, with TGD participants exposed to body neutrality having commented positively, including one non-binary individual stating “*My therapist recently introduced me to the idea of body neutrality, and I’ve felt a lot better about trying to reach that as a goal rather than body positivity … For me, coming to peace with my body makes more sense right now than diving head first towards love*” (66). Hence, body neutrality may offer a more manageable goal in the psychological shift away from a negative body image for those struggling with gender dysphoria.

Body neutrality is not without its critics in TGD communities, as many may view the ability to disregard the body’s importance as a privilege. Indeed, TGD individuals cannot always be neutral about their bodies, as presenting as one’s gender is not only helpful in stabilizing identity but is also important in feeling safe (69). Transgender individuals are over four times more likely than cisgender people to be the targets of violent crimes (70). When transgender people are perceived as cis-gendered, these crimes can be minimized. Therefore, being neutral about the body may not be as simple, especially when safety is at risk or when altering the body is a means to match identity. Therefore, while the no-judgement perspective of body neutrality may be more obtainable to TGD individuals than unconditional love of body positivity, other potential limitations may weaken body neutrality’s impact in these communities. Hence, research is needed to clarify the extent to which body-neutral and body-positive content are equally effective strategies in improving body image concerns for TGD TikTok users, especially in comparison to cis-gender TikTok users.

### 1.5 Current study

As TikTok continues to influence users’ body image perceptions, both body positivity and neutrality present distinct approaches to mitigating body dissatisfaction. Understanding these movements’ impact on users—especially on platforms driven by algorithms designed to maximize engagement—will be crucial in combating weight-stigmatization and promoting healthier body image ideals. Therefore, the aims of this study are three-fold: First, this study seeks to experimentally address whether viewing body-positive, body-neutral, and weight-stigmatizing content influences body dissatisfaction, mood, functional body appreciation, self-objectification, and appearance-related thoughts—and whether these impacts significantly differ. We predict that viewing body-positive and body-neutral content will be associated with overall better scores on all measures in comparison to weight-stigmatizing content. Additionally, because body positivity encourages a continued focus on appearance and may lead users to continue upholding a negative cognitive body image, whereas body neutrality steps away from appearance evaluations, we predict body-neutral content will be associated with higher levels of positive appearance thoughts and lower levels of body dissatisfaction and self-objectification compared to body-positive content. Additionally, we predict that body neutrality will result in higher functional appreciation than body positivity.

For our second aim, we will explore whether perceived body silhouette acts as a moderator in the relationship between content and body image outcomes. We make no specific hypotheses about how these moderation effects will manifest, as prior literature is extremely limited. Similarly, for our third aim, we will explore whether gender identity acts as a moderator in the relationship between content and body image outcomes. Again, we make no specific hypotheses about how these moderation effects will manifest, as prior literature is extremely limited.

## 2 Method

### 2.1 Participants

A sample of 326 adult participants who identified as women or gender diverse (e.g., transgender, non-binary, genderqueer), used TikTok, and lived within the United States were recruited via Connect Cloud Research, a professional participant platform. Data collection took place over a 13-day period in mid-2024, with participants receiving $3.50 following completion of the survey. Oversampling of participants with larger body types was performed to allow for moderation analyses with perceived body silhouettes. Similarly, gender diverse participants were also oversampled (cisgender women, 61.3%; transgender, non-binary, other 38.7%) for increased power regarding moderation analyses. Participants’ age ranged from 18–67 years (*M_age_* = 35.01, *SD* = 14.96) with the majority identifying as white (64.1%; Black or African American, 16.9%; Hispanic or Latino, 8.6%; Asian or Asian American, 8.6%; American Indian or Alaska Native, 1.5%; Native Hawaiian or Pacific Islander 0.3%) and heterosexual (49.4%; homosexual, 10.4%; bisexual, 29.1%; other, 11.0%). Participants’ most utilized social media platform was TikTok, with majority indicating daily use to be between 30 minutes to two hours (less than 30 minutes, 19.6%; 30 minutes - 1 hour, 23.0%; 1–2 hours, 23.0%; 2–3 hours, 21.8%; 3+ hours 12.6%). A comprehensive summary of the descriptive statistics is provided in **Supplementary Appendix A**: **Table A1**.

### 2.2 Design and procedure

The study was completed entirely online via Qualtrics with participants recruited through Connect. Because the study involved exposure to social media content that could elicit body dissatisfaction, participants were informed during the consent process that the study included scales and stimuli related to body image and social media (**Supplementary Appendix B**). The first part of the survey was a cross-sectional design consisting of questions regarding TikTok time usage, exposure to body-neutral and weight-stigmatizing content, self-esteem, and eating disorder (ED) behaviors and beliefs. The second part of the survey consisted of an experimental design in which participants were randomly assigned to one of four TikTok video conditions: body neutrality, body positivity, weight-stigmatizing, or travel. Participants completed measures of mood, body dissatisfaction, self-objectification, and functional appreciation pre- and post-exposure. Additionally, the frequency and positivity of thoughts about appearance as well as the likelihood to continue watching were assessed for each condition post-exposure. In case of potential distress, all participants were provided with a debriefing page at the end of the study, which included a list of resources for mental health and eating disorder support (Appendix B). The study took approximately 25 minutes to complete.

### 2.3 Stimulus materials

Four sets of TikTok video compilations based on body neutral, body positive, weight-stigmatizing, and travel were created by the researchers. In creating these conditions, TikTok reels for the two body acceptance conditions were initially searched by relevant hashtags (e.g. body neutral: #bodyneutrality, #bodyneutral; body positive: #bodypositivity, #bodypositive). Importantly, thematic analysis of content using body-neutral and body-positive hashtags have found some of the content to contain contradictory messages, such as promoting weight loss or praising thinness (49, 71–73). Hence, videos were selected based on how accurately they represented the ideas of body neutrality and body positivity. Additionally, to ensure there was no thematic overlap between the body-neutral and body-positive conditions, videos that featured themes or hashtags relating to both conditions, and body acceptance more generally, were not selected. Lastly, videos for both body acceptance conditions were selected to show a wide variety of creators of different ages, ethnicities, and body types.

Following this initial collection, the contents of the videos were then summarized and matched between conditions. Both conditions featured content explaining what body neutrality or body positivity is, mindfulness practices, songs, and the purpose of food, physical exercise, and/or clothes. Similarly, the selection of the weight-stigmatizing videos matched the content of the body acceptance conditions. For instance, videos on food for dieting purposes, physical exercise for weight loss, clothing for ‘flattering’ or ‘slimming’ purposes, and mantras encouraging thin idealization or promoting weight-stigma were selected, but excluded material that explicitly promoted disordered eating behaviors (e.g., binging, purging, extreme restriction). Creators in the weight-stigmatizing condition were all thin and/or lean. Lastly, videos for the travel condition were searched for using “#travel” and included content regarding travel destinations and scenic shots. Importantly, the creators’ bodies were not present in these videos.

Each of the four video compilations was piloted to ensure that the featured videos accurately reflected their corresponding categories. Participants in the pilot study (N = 17) were given a brief definition of their randomly assigned condition and asked to assess how well the videos represented that condition. Any videos identified as not representative of the condition were removed. Participants were also asked about the length of the compilations and ease of the manipulation check. The manipulation checks required participants to correctly identify a screenshot from the videos watched among two other photos featuring TikTok videos not shown. Following feedback from pilot participants, the video compilations for each condition were shortened to a total of five minutes, with an attention check prompted at the 2½-minute mark. Each five-minute compilation video consisted of approximately 20 videos, with an average length of 20 seconds.

### 2.4 Measures

#### 2.4.1 Demographics

Following consent, participants were asked demographic information including age, ethnicity, sexual orientation, education level, income, and relationship status (**Supplementary Appendix A**). Gender was assessed by asking people to indicate which gender they most identified out of the following options: Cisgender Woman, Cisgender Man, Transgender Woman, Transgender Man, Non-binary, and Other. General social media use was also assessed through the number of platforms used and the frequency of use for each platform from 1(Never) to 5 (Multiple times a day). Participants were also shown how to check their daily average TikTok time usage within the settings section of their phone and asked to report said number. Finally, to account for pre-existing engagement, participants’ prior exposure to body-positive, body-neutral, and weight-stigmatizing TikTok content was assessed using a modified version of the Body Positivity Media Exposure Scale (33), which included additional items for body-neutral and weight-stigmatizing themes based on Pellizzer and Wade (42).

#### 2.4.2 Body Silhouette

**Figure 1:**
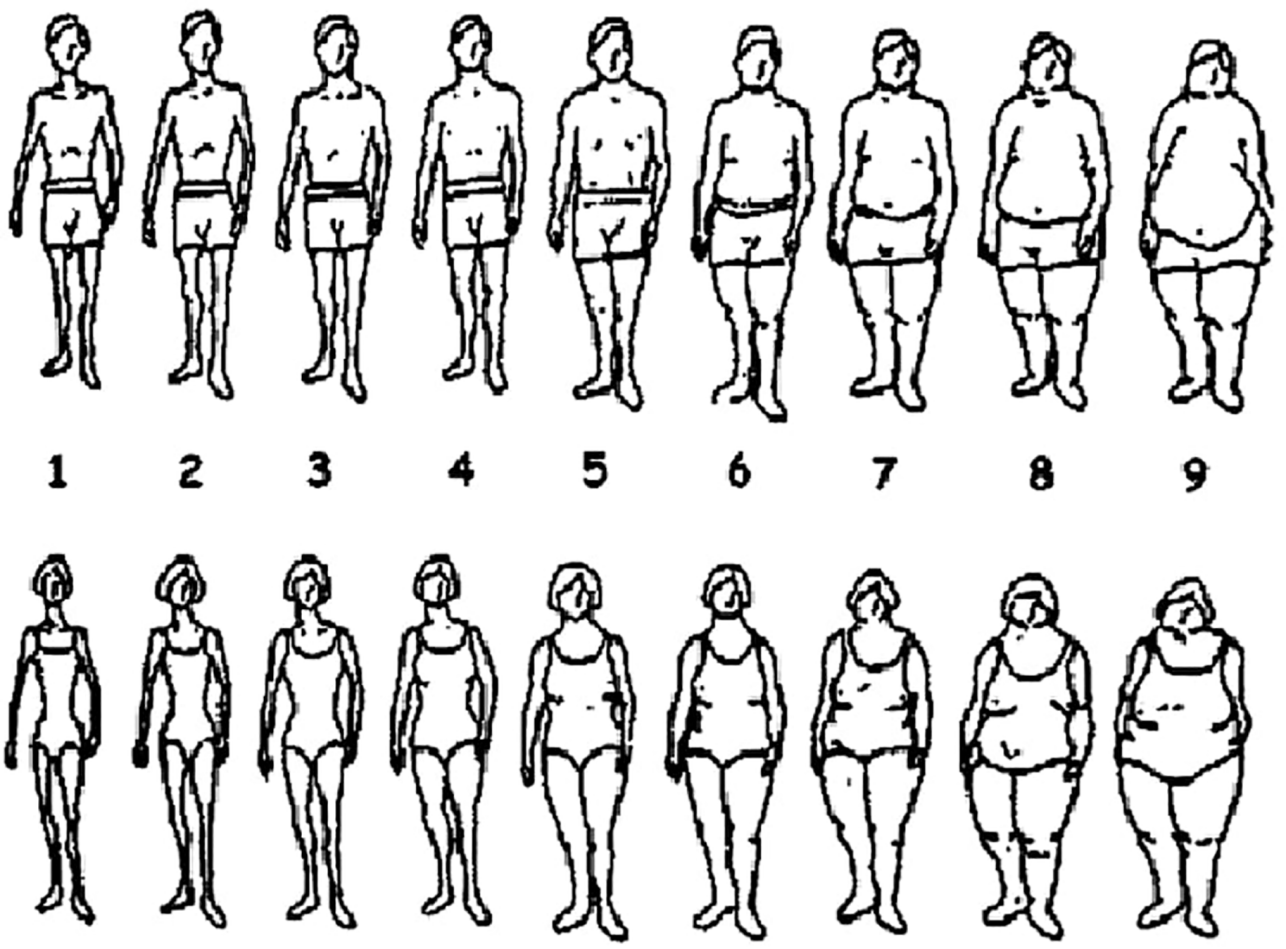
Stunkard figure rating scale (Stunkard, Sorensen, & Schulsinger, 1983).

The Stunkard figure rating scale, a visual scale that depicted 18 different figures, nine feminine and nine masculine presentation, and asked participants to “Indicate which silhouette you feel looks most like yourself” (74). See **Figure 1**.

#### 2.4.3 Pre and post measures

##### 2.4.3.1 Body dissatisfaction

Body dissatisfaction was assessed by asking participants to rate their current satisfaction on four dimensions: body shape, body size, weight, and appearance/attractiveness (75). Scales consisted of digital sliders ranging from 0 (No Dissatisfaction) to 100 (Very Dissatisfied). Scores were averaged across all four measures, with higher scores indicating higher levels of dissatisfaction. The scale showed very good reliability at both pre-exposure (α = .93) and post-exposure (α = .96).

##### 2.4.3.2 Mood

To assess mood the Positive and Negative Affect Scale (PANAS; 76), featuring 10 positive and 10 negative items, was utilized. Participants rated how much they were experiencing these feelings “right now” on a five-point scale ranging from not at all (0) to extremely (4). Means of the two subscales, positive and negative affect, were created (negative mood pre: α = .92; post: α = .91; positive mood pre: α = .92; post α = .95).

##### 2.4.3.3 Self-objectification

Self-objectification was assessed through the 7-item Self Objectification Beliefs and Behaviors Representing Self Subscale (77). This scale measures how much participants view themselves as an object to be evaluated based on their appearance, with an example item being “My physical appearance is more important than my personality.” Responses were measured along a 5-point scale from strongly disagree (-2) to strongly agree (2). Scores were averaged such that higher scores indicated higher levels of self-objectification. The scale showed very good reliability (pre: α = .92; post α = .95).

##### 2.4.3.4 Functional appreciation

To measure functional appreciation, the 7-item Functionality Appreciation Scale (FAS; 78) was administered. Functional appreciation is a form of positive non-appearance-based body image, in which participants indicate how appreciative they are of their body’s abilities. An example item read, “I respect my body for the functions that it performs.” Responses were measured along a 5-point scale from strongly disagree (-2) to strongly agree (2), with higher scores indicating higher levels of functionality appreciation. The scale showed very good reliability at pre-test (α = .92) and post-test (α = .95).

#### 2.4.4 Post measures

##### 2.4.4.1 State appearance thoughts

Appearance-related thoughts during the video conditions were assessed using two items. The first item assessed the frequency of such thoughts by asking participants, “While watching the videos, to what extent did you think about your own appearance?” However, as appearance-related thoughts are not inherently negative, a second item evaluated their valence: “To what extent were any thoughts about your appearance positive?” Both items were rated on a 5-point scale, ranging from none at all (0) to a great deal (4).

##### 2.4.4.2 Likelihood to continue watching

We also wanted to assess participants’ likelihood in continuing to watch body neutrality and body positivity outside of study conditions. To assess such, participants were asked “To what extent do you feel you would like to continue viewing or following the content you watched?” Responses were ranked on a 5-point scale from not at all (0) to a great deal (4).

### 2.5 Data analysis

Participants that were missing significant data (>50%), failed more than 2 out of 3 attention checks, or bypassed the condition criteria were removed, resulting in a final sample size of N = 326. A sensitivity analysis was conducted using G*Power (79) to determine the minimum detectable effect size for repeated measures within-between interaction with a sample size of 326. Assuming an alpha level of.05 and a power (1 – β) of.80 the analysis indicated that the minimum detectable effect size was f = 0.09, corresponding to a partial η² = .18 (small effect; 80). Thus, our final sample size of 326 was adequate to detect small effect sizes. An available item analysis was conducted to address any additional missing data points, leading to slight variations in the number of participants included in each analysis. To test hypothesis 1, mixed repeated-measures ANOVAs were conducted to examine condition differences over time for body dissatisfaction, functional appreciation, self-objectification, and positive affect and negative affect. An exploratory ANCOVA was also conducted with pre-exposure scores as a covariate to control for individual differences at pretest, which may increase sensitivity to detect group-level differences that were not apparent in the unadjusted comparisons. For measures assessed only after condition exposure—specifically, frequency of appearance-related thoughts, positivity of appearance-related thoughts, and likelihood of continuing to watch—the scores were entered into a separate MANOVA. All *post hoc* pairwise comparisons utilized the Bonferroni test to reduce risk of a Type 1 error.

## 3 Results

### 3.1 Preliminary analyses

Chi-square tests and a MANOVA were conducted to ensure no initial differences across the four experimental conditions. There were no significant condition differences in age, race, education, relationship status, sexuality, region, urbanicity, income, body silhouette, total social media accounts, overall frequency of social media use, time spent on TikTok, previous exposure to body acceptance content, and previous exposure to weight-stigmatizing content. There were also no significant condition differences in pre-exposure scores for body dissatisfaction, functional appreciation, self-objectification, positive affect, and negative affect. Means and standard deviation scores for participants in each condition on each of the outcome measures at each time are reported in **Table 1**.

**Table 1:** Mean and standard deviations for TikTok content conditions on outcome variables.

Variable	Scale Range	Body Positive (n=81)		Body Neutral (n=82)		Thin Ideal (n=79)		Travel (n=84)	
		*M*	*SD*	*M*	*SD*	*M*	*SD*	*M*	*SD*
Body Dissatisfaction	1 - 100								
Pre-exposure		53.75	29.16	58.68	28.01	58.06	26.94	54.01	31.28
Post-exposure		45.72*_b_	30.71	51.26*_b_	29.40	61.10_a_	30.83	49.54*_b_	32.14
Functional Appreciation	-2 - 2								
Pre-exposure		1.09	0.73	1.04	0.74	0.98	0.86	0.94	0.79
Post-exposure		1.25*_a_	0.80	1.18*_a_	0.69	0.91_b_	0.98	0.99_ab_	0.82
Self-Objectification	-2 - 2								
Pre-exposure		-0.86	0.91	-0.69	1.00	-0.76	1.02	-0.79	1.04
Post-exposure		-0.79*_ab_	1.04	-0.90*_b_	1.02	-0.72_a_	1.14	-0.91_ab_	1.03
Positive Affect	0 - 4								
Pre-exposure		1.68	1.02	1.58	0.88	1.54	0.87	1.54	0.94
Post-exposure		1.81*_a_	1.13	1.68_a_	0.97	1.20*_b_	1.04	1.62_a_	1.08
Negative Affect	0 - 4								
Pre-exposure		0.58	0.68	0.62	0.74	0.56	0.73	0.65	0.79
Post-exposure		0.38*_b_	0.53	0.42*_b_	0.63	0.70*_a_	0.80	0.40*_b_	0.60
Post-Test Only Variables	0 - 4								
View Likelihood		1.93_a_	0.13	1.54_a_	0.13	0.93_b_	0.14	1.76_a_	0.13
Appearance Frequency		2.41_a_	0.12	2.41_a_	0.12	2.31_a_	0.12	0.72_b_	0.12
Appearance Positivity		2.18_a_	0.12	2.05_a_	0.12	1.22_b_	0.97	1.29_b_	0.12

*significant difference between pre and post scores at p < .05.

Different subscripts indicate a significant difference between conditions at p < .05, based on ANCOVA results.

### 3.2 Body dissatisfaction

**Figure 2:**
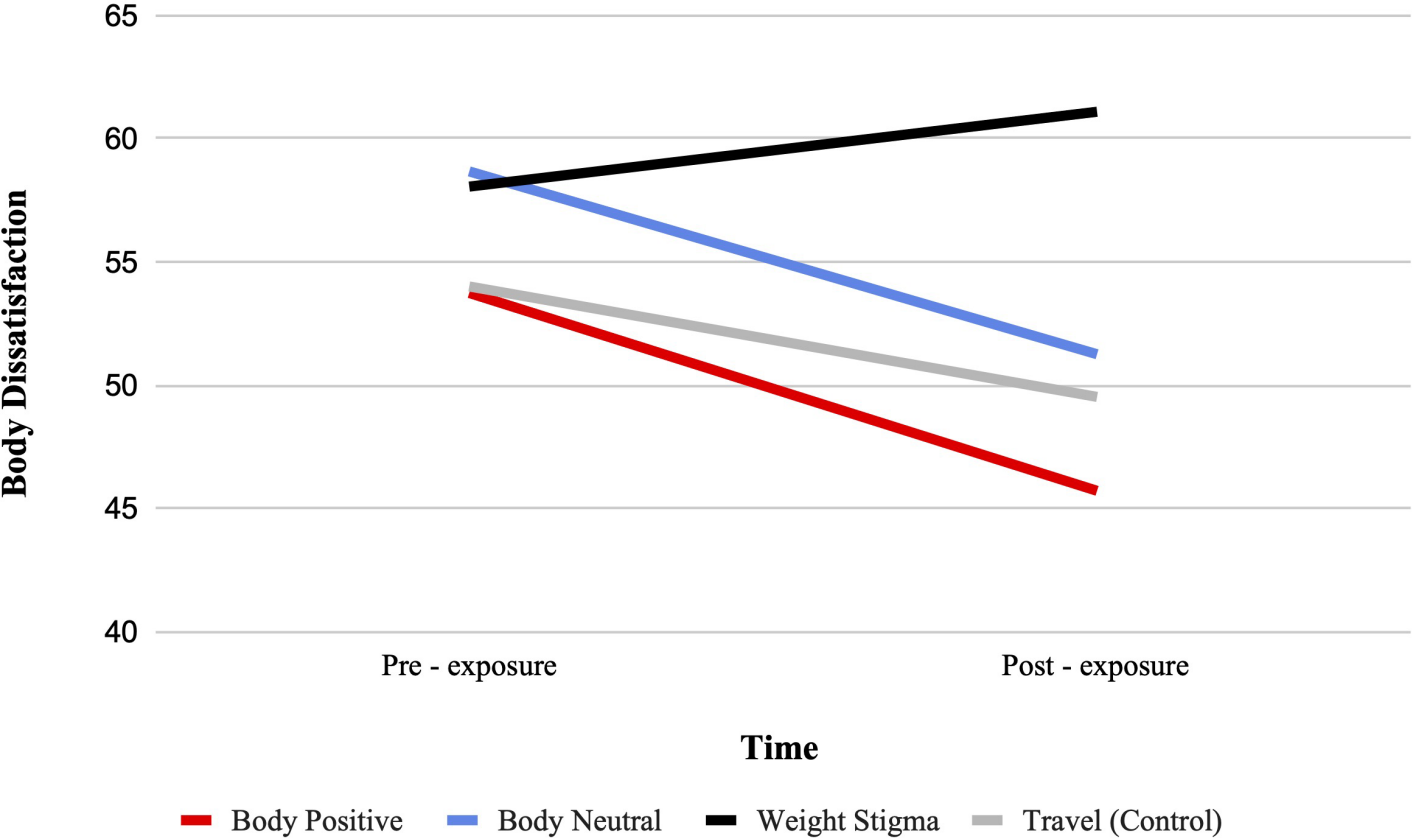
Estimated marginal means for body dissatisfaction. A representation of the interaction between condition and time for body dissatisfaction, such that higher scores indicate higher body dissatisfaction.

There was a significant condition by time interaction for body dissatisfaction from pre-test to post-test, F (3, 321) = 8.75, p <.001, ηp2 = .08. See **Figure 2**. Bonferroni-adjusted pairwise comparisons showed that body dissatisfaction significantly decreased from pre- to post-test in the body positive condition (MD = -8.03, SD = 1.71, p <.001), the body neutral condition (MD = -7.42, SD = 1.70, p <.001), and the travel condition (MD = -4.47, SD = 1.68, p = .008). No significant change was observed in the weight stigma condition.

Between-group comparisons at post-test indicated that body dissatisfaction was significantly lower in the body positive condition compared to the weight-stigma condition (MD = 15.39, SD = 4.87, p = .01), with no other significant differences observed. The ANCOVA result showed a significant main effect of video condition, F(3, 320) = 8.14, p <.001, ηp2 = .071, with the body positive (MD = 10.86, SD = 2.38, p <.001), body neutral (MD = 9.41, SD = 2.38, p = .001), and travel (MD = 7.14, SD = 2.36, p = 0.016) condition demonstrating significantly lower body dissatisfaction than the weight-stigma condition.

### 3.3 Functional appreciation

**Figure 3:**
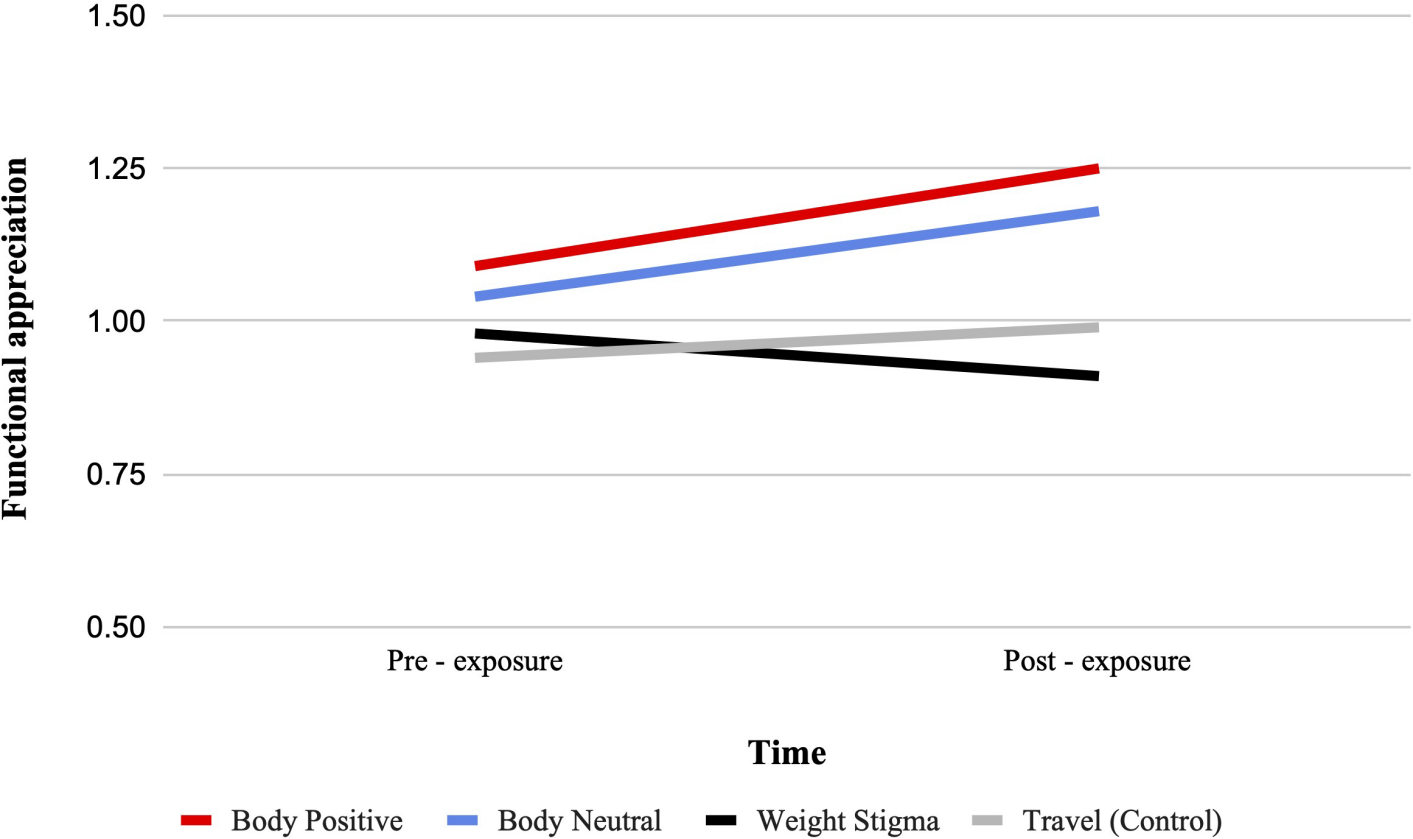
Estimated marginal means for functional appreciation. A representation of the interaction between condition and time for functional appreciation, such that higher scores indicate higher functional appreciation.

There was a significant condition by time interaction for functional appreciation from pre-test to post-test, F (3, 322) = 5.02, p = .002, ηp2 = .045. See **Figure 3**. Bonferroni-adjusted pairwise comparisons showed that functional appreciation significantly increased from pre- to post-test in the body positive condition (MD = 0.16, SD = 0.05, p <.001), and the body neutral condition (MD = 0.14, SD = 0.05, p = .003). No significant change was observed in the weight stigma or travel condition.

Between-group comparisons at post-test indicated that functional appreciation was marginally higher in the body positive condition compared to the weight-stigma condition (MD = 0.034, SD = 0.13, p = .05), with no other significant differences observed. However, an ANCOVA with pre-exposure scores as a covariate found a significant main effect of video condition, F(3, 321) = 5.55, p = .001, ηp2 = .049, with both the body positive (MD = 0.24, SD = 0.07, p = .002) and body neutral (MD = 0.21, SD = 0.07, p = .007) condition having significantly higher functional appreciation than the weight-stigma condition.

### 3.4 Self-objectification

**Figure 4:**
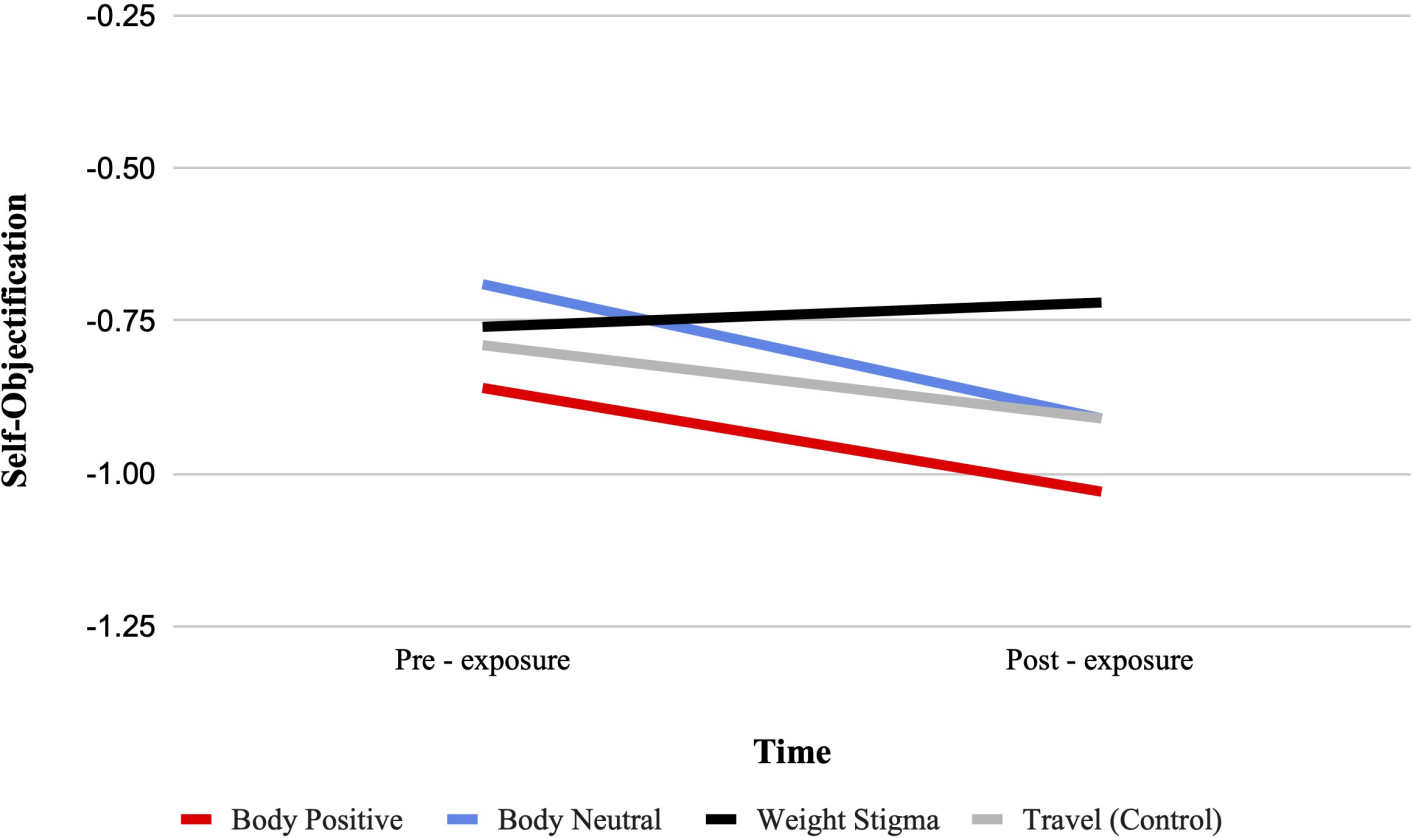
Estimated marginal means for self-objectification. A representation of the interaction between condition and time for self-objectification, such that higher scores indicate higher self-objectification.

There was a significant condition by time interaction for self-objectification from pre-test to post-test, F (3, 322) = 2.82, p = .039, ηp2 = .026. See **Figure 4**. Bonferroni-adjusted pairwise comparisons showed that self-objectification significantly decreased from pre- to post-test in the body positive condition (MD = -0.17, SD = 0.07, p = .010), and the body neutral condition (MD = -0.22, SD = 0.07, p = .001). No significant change was observed in the weight stigma or travel condition.

Despite a significant interaction, between-group comparisons at post-test indicated no significant differences between conditions. However, an ANCOVA with pre-exposure scores as a covariate found a significant main effect of video condition, F (3, 321) = 2.94, p = .034, ηp2 = .027, with the body neutral condition (MD = 0.25, SD = 0.09, p = .045) having significantly lower self-objectification than the weight-stigma condition.

### 3.5 Mood

#### 3.5.1 Positive affect

**Figure 5:**
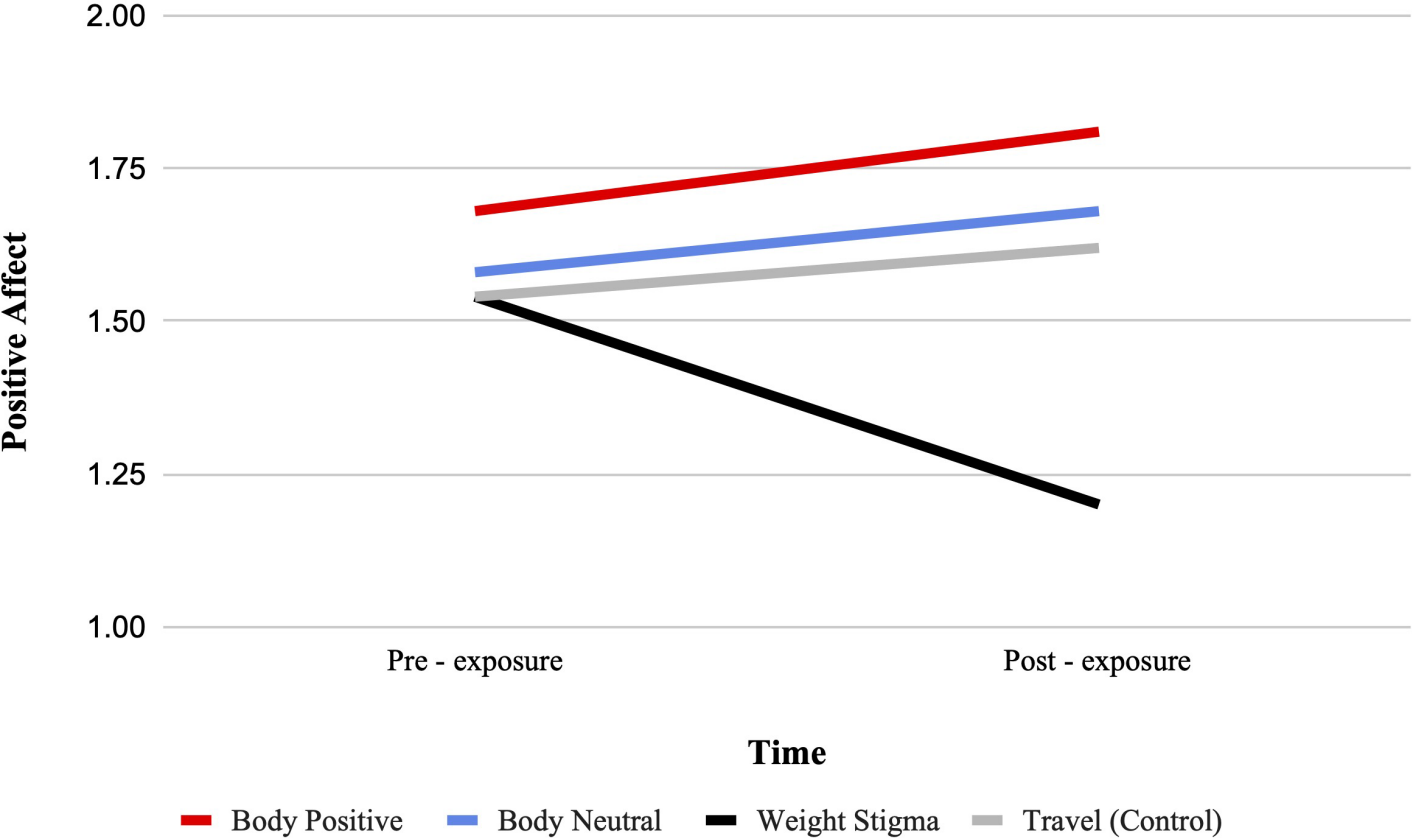
Estimated marginal means for positive affect. A representation of the interaction between condition and time for positive affect, such that higher scores indicate higher positive affect.

There was a significant condition by time interaction for positive affect from pre-test to post-test, F (3, 322) = 11.51, p <.001, ηp2 = .097. See **Figure 5**. Bonferroni-adjusted pairwise comparisons indicated that positive affect marginally increased from pre- to post-test in the body positive condition (MD = 0.13, SD = 0.07, p = .051) and a significant decrease from pre- to post-test in the weight stigma condition (MD = 0.35, SD = 0.07, p <.001). No significant change was observed in the body neutral or travel condition.

Between-group comparisons at post-test indicated that positive affect was higher in the body positive (MD = 0.61, SD = 0.17, p = .002) and body neutral (MD = 0.49, SD = 17, p = .022) condition compared to the weight-stigma condition, with no other significant differences observed. The ANCOVA with pre-exposure scores as a covariate similarly found a significant main effect of video condition, F(3, 321) = 11.77, p <.001, ηp2 = .099, but the body positive (MD = 0.49, SD = 0.09, p <.001), body neutral (MD = 0.46, SD = 0.09, p <.001), and travel (MD = 0.43, SD = 0.09, p <.001) condition all showed significantly higher positive affect than the weight-stigma condition.

#### 3.5.2 Negative affect

**Figure 6:**
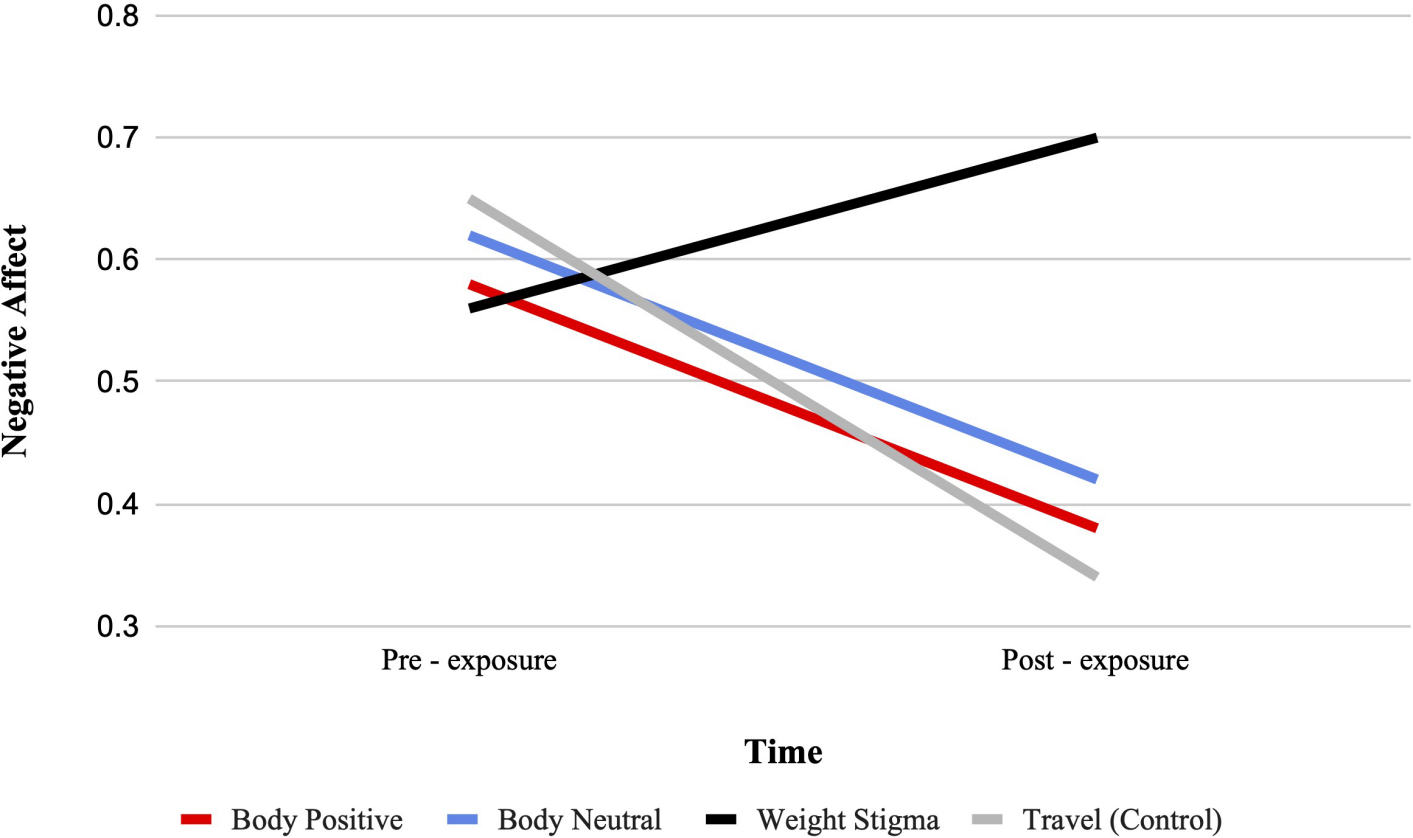
Estimated marginal means for negative affect. A representation of the interaction between condition and time for negative affect, such that higher scores indicate higher negative affect.

There was a significant condition by time interaction for negative affect from pre-test to post-test, F (3, 322) = 9.90, p <.001, ηp2 = .084. See **Figure 6**. Bonferroni-adjusted pairwise comparisons indicated significant changes in negative affect from pre- to post-test for all conditions. Specifically, negative affect decreased in the body positive (MD = -0.20, SD = 0.06, p <.001), body neutral (MD = -0.20, SD = 0.06, p <.001), and travel condition (MD = -0.25, SD = 0.06, p <.001) while increased in the weight-stigma condition (MD = 0.14, SD = 0.06, p = 0.16).

Between-group comparisons at post-test indicated that negative affect was lower in the body positive (MD = 0.32, SD = 0.10, p = .013), body neutral (MD = 0.28, SD = 10, p = .038), and travel condition (MD = 0.30, SD = 0.10, p = .019) compared to the weight-stigma condition, with no other significant differences observed. The ANCOVA with pre-exposure scores confirmed these results with a significant main effect of video condition, F(3, 321) = 11.82, p <.001, ηp2 = .099, with body positive (MD = 0.33, SD = 0.07, p <.001), body neutral (MD = 0.32, SD = 0.07, p <.001), and travel (MD = 0.36, SD = 0.07, p <.001) condition having significantly lower negative affect than the weight-stigma condition.

### 3.6 Frequency and positivity of appearance thoughts

There was no difference in frequency of appearance thoughts between body positive, body neutral, or weight-stigmatizing condition. Critically, however, participants reported that these appearance thoughts were more positive in the body positive (p <.001) and body neutral (p <.001) conditions than in the weight-stigma condition. Furthermore, participants indicated they were more likely to continue watching body positivity (p = .009) and body neutrality (p <.001) in comparison to the weight-stigmatizing condition. Participants in the travel condition did significantly think about their appearance less often than in the other three conditions (p<.001); however, frequency of positive thoughts were significantly lower than both the body neutral (p <.001) and body positive condition (p <.001).

### 3.7 Gender identity and body silhouette

To examine whether gender identity moderated the effects of video condition on body image and mood outcomes, we conducted 2 (time: pre, post) x 4 (condition: body neutral, body positive, weight-stigma, travel) x 2 (gender: cisgender, transgender/gender diverse) mixed repeated-measure ANOVAs. There was a significant main effect of gender on body dissatisfaction, F(1, 313) = 4.56, p = .034, ηp2 = .01, self-objectification, *F*(1, 313) = 8.34, *p* = .004, ηp² = .03, and positive affect, *F*(1, 313) = 12.66, *p* <.001, ηp² = .04, indicating that transgender and gender diverse participants reported lower body dissatisfaction, greater self-objectification, and lower positive affect overall than cisgender participants. There was also a significant main effect of gender identity on likelihood to continue watching, with TGD participants indicating they were less likely to continue watching any of the featured content than cisgender participant, F (1, 313) = 25.53, p <.001, ηp² = 08.

Gender identity was not involved in any significant two-way or three-way interactions with time or video condition for any outcomes within the repeated measure ANOVAs. However, a marginally significant three-way interaction emerged for body dissatisfaction within the ANCOVA analyses, F (3, 312) = 2.52, p = .058, ηp² = .02. Exploratory follow-up analyses revealed that while body positivity was equally effective across gender identities, body neutrality was associated with significantly lower body dissatisfaction for transgender and gender-diverse individuals compared to cisgender participants (p = .029).

To examine whether body silhouette moderated the effects of video condition on body image and mood outcomes, we similarly conducted a series of 2 (time: pre, post) × 4 (condition: body neutral, body positive, weight-stigma, travel) × 3 (body silhouette: smaller bodied [1–3], mid bodied [4–6], larger bodied [7–9]) mixed repeated-measures ANOVAs. We grouped body silhouettes into three categories to capture meaningful differences in body size while maintaining statistical power. In other words, this categorization balances nuance and power, as larger groupings would compromise statistical validity while broad groupings (e.g., 1–4 vs. 5-9) would obscure important nuances. There was a significant main effect of body silhouette on body dissatisfaction, F(2, 314) = 21.66, p <.001, ηp² = .12, such that participants with smaller body silhouettes reported significantly lower body dissatisfaction than those with mid-sized (p <.001) and larger silhouettes (p <.001), and mid-sized participants reported lower dissatisfaction than larger-bodied participants (p = .02). There was also a significant main effect of body silhouette on positive affect, F (2, 314) = 3.17, p = .043, ηp² = .02. *Post hoc* comparisons revealed that mid-sized participants reported significantly higher positive affect than larger-bodied participants (p = .035), while no significant differences were found between the smaller and mid-sized or smaller and larger silhouette groups.

Body silhouette was not involved in any significant two-way or three-way interactions with time or video condition for body image or negative affect. However, a marginally significant three-way interaction emerged for positive affect, F (6, 314) = 2.06, p = .058, ηp² = .04. Exploratory follow-up analyses revealed that, within the body positive condition, only participants with mid-sized bodies showed a significant increase in positive affect from pre- to post-exposure (p <.05). In the body neutral condition, participants with larger bodies demonstrated a marginal increase in positive affect (p = .058). In contrast, in the weight-stigma condition, mid- and larger-bodied participants experienced a significant decrease in positive affect (p <.05), whereas no significant changes were observed for participants with smaller bodies in any condition. Notably, the ANCOVA analyses did not replicate these findings, as no significant interactions were observed.

## 4 Discussion

Our study was one of the first to our knowledge to compare the differential impacts of body positivity and body neutrality on body image and mood. Furthermore, it is the first to our knowledge to compare body positivity and body neutrality across different gender identities and body silhouettes. Accordingly, the aim of our study was three-fold: (i) investigate the effect of brief exposure to TikTok body neutral, body positive, and weight-stigmatizing content on functionality appreciation, body dissatisfaction, self-objectification, and mood in women and gender diverse users; (ii) to explore whether perceived body silhouette acts as a moderator in the relationship between content and its impacts; and (iii) to explore whether gender identity acts as a moderator in the relationship between content and its impacts. Below we discuss the main findings, implications, and future directions.

The results provided partial support for our hypothesis that body-acceptance content on TikTok leads to improved body image and mental health, with significant improvements to functional appreciation, self-objectification, body dissatisfaction, and negative affect. In addition, although participants in all experimental conditions reported thinking about their appearance to a similar extent during exposure, those in the body-positive and body-neutral conditions reported more positive thoughts compared to those in the weight-stigmatizing and travel conditions. Interestingly, analysis of significant differences between conditions at time 2, following exposure, differed between the repeated-measures ANOVAs and ANCOVAs. While similar results for body positivity and body neutrality were obtained regarding positive and negative affect, the ANCOVAs showed a broader set of significant differences. Specifically, the ANCOVAs found that both body positivity and body neutrality significantly differed from weight stigma on body dissatisfaction and functional body appreciation. Additionally, body neutrality was the only condition to significantly differ from weight stigma on self-objectification. This may suggest that controlling for individual baseline differences may have clarified effects that were masked by within-subject variability in the rmANOVA. As such, the ANCOVA results may more accurately reflect the unique contribution of each condition after accounting for individual differences. Finally, resulted indicated marginal three-way interactions suggested that the effects of condition on body dissatisfaction varied by gender identity, and effects on positive affect varied by body silhouette.

Hence, the present study contributes to the limited existing body-neutral and body-positive literature through multiple novel findings. Our findings suggest that brief exposure to either body-positive or body-neutral TikTok content can lead to similar improvements overall in functional appreciation, body dissatisfaction, and negative mood. These findings align with the existing literature exploring body-neutral (59, 60) and body-positive content on social media (40, 48, 81), suggesting the potential for TikTok to foster growth in body image and mood depending on content viewed. That being said, while our study confirmed our hypothesis and aligned with previous literature by showing that weight-stigmatizing content significantly worsened positive mood and body positivity marginally improved positive mood, body neutrality had no effect, which was unexpected. This may suggest that body positivity content is more effective at improving positive affect compared to body neutrality. Alternatively, this discrepancy may be due to the proportion of high versus low arousal positive emotions featured within the PANAS. The positive emotions assessed are primarily high arousal emotions, or feelings that are more intense and energetic, including excited, enthusiastic, alert, and attentive. Low arousal emotions, or emotions relating to feelings of being subdued and relaxed (e.g. confident, content) are underrepresented, with only one positive low arousal emotion in the scale (i.e. ‘interested’). Previous studies that found body acceptance content to enhance positive mood tended to have a higher ratio of low- to high-arousal emotions (40, 48, 81). In contrast, studies that did not observe improvements, or found improvements comparable to the control condition, reported a lower low- to high-arousal ratio (45, 59). Hence, body acceptance content, partially body neutrality, may be improving low arousal positive mood rather than high arousal positive mood. Given the marginal significance of the current findings, replication with larger samples and more nuanced measures of arousal is needed to clarify the role of positive affect. Future research should further explore this by directly comparing body positivity and neutrality conditions on measures of both high- and low-arousal positive emotions.

Alternately, our exploratory moderation analysis found that improvements in positive mood following body positivity exposure were limited to participants with mid-sized silhouettes, while improvements were only marginally present in those with larger bodies post body neutral exposure. Alongside suggesting that body positivity and body neutrality content may not universally benefit all body sizes, this may also partially explain the overall null effects on positive mood: improvements for some subgroups (e.g., mid-sized individuals) may have been obscured by the lack of change among others. Future research should explore how body size moderates the mood impact of body acceptance content, and whether high vs low arousal emotions are differentially influenced by body size and exposure to body acceptance content. Despite these exploratory findings, it is important to note that our hypothesized moderation effects were not supported across the full model. Specifically, body silhouette did not significantly moderate the relationship between video condition and time for most outcomes, and no moderation effects were observed in the exploratory ANCOVA analyses. As such, these subgroup trends should be interpreted with caution, as they were not consistently replicated across analytic approaches or the model. Future research should aim to replicate these preliminary patterns and test more targeted models to better understand for whom and under what conditions body acceptance content is most effective.

Building on the improvements observed across conditions, we next explored significant differences between conditions at time 2, following exposure. Contrary to our hypothesis, the results revealed limited differences between the body acceptance conditions, with the only significant distinction between body positivity and body neutrality occurring in relation to self-objectification. Specifically, while participants in the body neutral condition significantly differed from the weight-stigmatizing condition on improvements in self-objectification, the body positivity condition did not differ from the weight-stigmatizing condition on self-objectification changes. This finding supports prior research showing that while both movements may be beneficial in improving body image, body positivity’s reliance on appearance potentially limits one’s ability to decrease self-objectification beliefs (49, 58).

Despite these limited differences between conditions overall, ANCOVA moderation analyses did reveal marginally significant differences in body dissatisfaction based on gender identity. Specifically, while body positivity was equally effective across gender identities, body neutrality was associated with significantly lower body dissatisfaction for transgender and gender-diverse individuals compared to cisgender participants. This suggests that body neutrality may be more effective for transgender individuals than for cisgender individuals. Although not directly comparable, this is supported by previous literature that suggested body neutrality is not only effective within TGD community (60), but may be a more manageable goal than body positivity for those struggling with gender dysphoria (68). However, given the marginal significance of these findings, and no significant difference between body positivity and body neutrality for either cisgender or TGD participants, results should be interpreted with caution.

Finally, participants in both the body-positive and body-neutral conditions did not significantly differ from the travel condition. Participants in the travel condition showed significant improvements to body dissatisfaction and negative affect, but not positive affect, self-objectification, or functional appreciation. These results may suggest that appearance-neutral (aka non-body) content could give participants a respite from intrusive thoughts and comparisons about their body, thus leading to improved body satisfaction and mood. Our findings align with previous research demonstrating that appearance-neutral content not only reduces body dissatisfaction and negative mood but also elicits fewer upward appearance comparisons than both weight-stigmatizing and body acceptance content (75, 82, 83). While we did not specifically assess upward comparisons, participants in the travel condition reported fewer appearance-related thoughts than those exposed to weight-stigmatizing and body acceptance content. This suggests even positively framed body acceptance content may inadvertently trigger appearance comparisons in some users (33, 40, 50, 59), although to a lesser extent than weight-stigmatizing content. In other words, appearance-neutral content may serve as a beneficial alternative to body centered content for those most vulnerable to appearance comparisons with future studies needing to explore these effects further. However, avoiding body-centered content may not be practical or desirable for many users who report enjoying videos relating to body-centered themes (e.g. fashion, physical exercise, food). In these instances, recommending body-positive or body-neutral alternatives to typical weight-stigmatizing content would be a better solution than suggesting users avoid body topics altogether.

### 4.1 Practical and clinical implications

The present findings hold important theoretical and practical implications. Our findings, taken together with previous research, suggest that body positivity and body neutrality may allow TikTok users to foster positive relationships with their body and improve negative mood, potentially counteracting the effects of internalized weight stigma. With users beginning to call for alternative content to harmful body content, our findings suggest that body-neutral and body-positive content may serve as accessible and appealing public health strategies to reduce weight stigma in online settings. Indeed, recommendations for individuals to seek out body positivity instead of weight-stigmatizing content to protect their body image and mood have already been made by researchers (e.g., 47, 81). Expansion of these recommendations to include body neutrality is also needed. These findings partially underscore the importance of comprehensive social media literacy programs for youth, given increased impressionability and risk for development of body dissatisfaction, weight stigma beliefs, and disordered eating. Beyond teaching users how to identify harmful and stigmatizing content, these programs should include extensive education on body positivity, body neutrality, and fostering a positive relationship with the body. Equipping young users with these skills can empower them to critically assess the media they consume and mitigate the adverse effects of exposure to harmful thin idealizing and weight stigmatizing content (40, 71, 84, 85).

However, it is difficult for an individual to overcome the TikTok algorithms; thus, it is incumbent upon TikTok to alter their business practices to combat weight-stigmatizing content for the benefit of their users. This solution could be as simple as making users more aware of features that already exist within the platform, such as the ability to block or follow certain hashtags, set screen time limits, and prompted breaks, as well as more extensive initiatives that restructure the content in which users are exposed to. With participants indicating they were more interested in continuing to watch body-positive and body-neutral content than weight-stigmatizing content, it would be recommended for TikTok to alter their algorithms to push more body-positive and body-neutral content to users. In addition, TikTok could leverage artificial intelligence to flag, label, and filter out harmful content—particularly content related to diet and weight loss that may contribute to negative body image and weight stigma. Other measures include labeling videos as edited, allowing users the option to opt out of advertisements related to diet and weight loss-related products, or incorporate proactive prompts that encourage users to take a break from body-focused content, check in with themselves, and introduce healthier content alternatives.

### 4.2 Limitations

The current study should be interpreted within the context of several limitations. First, exposure to video conditions was 5 minutes; hence, the effects are short-term, and duration is unknown. Future studies should not only examine the persistence of these short-term effects but also investigate the potential cumulative impact of long-term exposure to body-positive and body-neutral content. Furthermore, given that our findings did not support the hypothesis that body positive and body neutral content significantly improves positive mood following brief exospore, a longitudinal study could help determine whether repeated exposure over several weeks or months is required for significant improvements. Second, while our sample was diverse regarding body type, sexuality, gender identity, and age, future studies should explore more diverse samples regarding race and disability status. Third, the videos utilized within the body-acceptance conditions were selected to portray the themes of body positivity and body neutrality while also featuring a wide variety of identities. However, it is well known that even with body acceptance spaces, weight-stigma and exclusionary practices occur (49). Further studies exploring the impacts of body acceptance content as it naturally exists within social media spaces are needed.

Additionally, while the lack of significant moderation effects could suggest that body acceptance content is broadly effective across gender and body silhouette—except for body dissatisfaction and positive affect respectively—there are several important limitations to consider regarding the moderation analyses. First, the body silhouette scales used in this study have received criticism for lacking diversity in body types and reinforcing binary gender representations. Although we attempted to reduce binary limitation by presenting all participants with both male and female silhouettes, the binary framing and limitation of only nine body types may still have influenced how participants engaged with the measure. Second, the body-positive, body-neutral, and the weight stigmatizing conditions exclusively featured feminine presenting creators who identified as women, though their gender identities were not explicitly stated to viewers. Although transgender individuals may have been included, their identities were not explicitly indicated, potentially limiting relevancy for gender-diverse participants. This limitation may have been reflected in our findings, given gender-diverse participants were significantly less likely than cisgender participants to indicate they would continue watching any of the content. Lastly, statistical power was limited. Although we had 126 gender-diverse participants and 71 participants with larger body sizes, distributing these participants across four video conditions resulted in relatively small subgroups, potentially limiting our ability to detect moderation effects. Future studies should aim to use more inclusive and representative measures and content, while ensuring sufficient power to test for nuanced identity effects.

## 5 Conclusion

Taken together, this study provides preliminary evidence that viewing body-positive and body-neutral TikTok content can lead to an improvement in body image and negative affect after only a brief 5-minute exposure. Given the high number of participants expressing interest in continuing to watch body acceptance content, both body neutrality and body positivity appear to be promising and feasible initiative in response to the growing demand for content that promotes body acceptance and challenges weight stigma.

## Data Availability

The datasets for this study can be found at https://osf.io/8q2xj/.
