# Supplementary material for: Combating weight-stigmatization in online spaces: the impacts of body neutral, body positive, and weight-stigmatizing TikTok content on body image and mood
Source: Front Psychiatry. 2025 Jun 10;16:1577063. doi: 10.3389/fpsyt.2025.1577063 (PMC12185992; doi:10.3389/fpsyt.2025.1577063)
Supplement: Supplementary file 2 [file SupplementaryFile2.docx]

**Appendix A: Demographics**

**
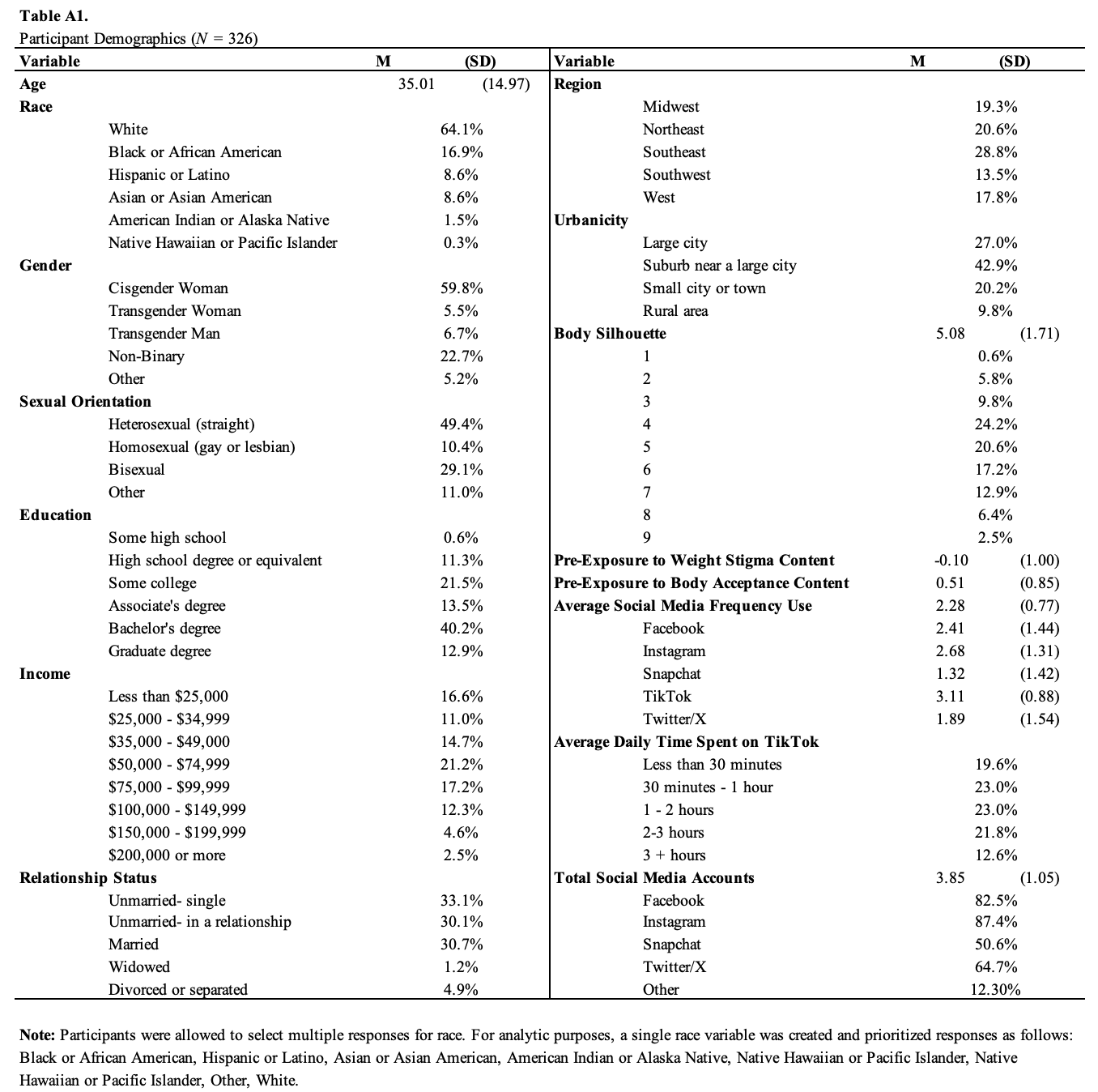
**

**Appendix B: Consent and Debriefing**

**CONSENT FORM**

  We are researchers in the School of Social & Behavioral Sciences at Arizona State University. We are conducting research investigating social media behaviors and wellbeing. We are inviting your participation, which will involve approximately 15-20 minutes to complete an online survey including questions and stimuli about basic demographic information, social media use, body dissatisfaction, and well-being.

 The survey is only applicable to individuals who identify as females or gender diverse (non-binary, transgender, etc.) and use TikTok. You must speak English, live in the United States, and be 18 or older to participate in the study.

 You have the right not to answer any question, and to stop participation at any time. Your participation in this study is voluntary. If you choose not to participate or to withdraw from the study at any time, there will be no penalty. You will be compensated approximately $3.00 for completing the survey. This study consists of a one-time survey. Your participation will help us understand how virtual interactions can influence individuals’ behaviors and mental health.

There are no major foreseeable risks or discomforts to your participation. Data will be stored on encrypted ASU secure servers. The files will be password protected. We will not ask your name or any other identifying information in this survey. For research purposes, an anonymous numeric code will be assigned to your responses. Your Connect worker ID number will be temporarily stored in order to pay you for your time; this data will be deleted as soon as it is reasonably possible. You have the option of making your personal information private by changing your Connect. 

 The results of this study may be used in reports, presentations, or publications but your name will not be used. The results will only be shared in the summary form. If you have any questions concerning the research study, please contact the Principal Investigator. If you have any questions about your rights as a subject/participant in this research, or if you feel you have been placed at risk, you can contact the Chair of the Human Subjects Institutional Review Board, through the ASU Office of Research Integrity and Assurance, at (480) 965-6788. By selecting “AGREE” below you are agreeing to be part of the study.

- **AGREE** (4)

**DEBRIEFING EXCERPT**

Thank you so much for your participation in this study!

If you have any questions or concerns, you can contact the principal investigator: (*email was provided*)

If you or anyone you know needs additional support regarding mental health or body image issues, please refer to the resource below:

**https://www.mentalhealth.gov/get-help/immediate-help**

**Substance Abuse and Mental Health Services Administration (SAMHSA)**

Find Help Website: https://www.samhsa.gov/find-help/national-helpline

Helpline Phone Number: 1-800-662-HELP (4357); (24/7, 365 days/year, confidential, free)

**National Eating Disorders Association (NEDA)**

Website: https://www.nationaleatingdisorders.org/.

Helpline Info: https://www.nationaleatingdisorders.org/help-support/contact-helpline.

Helpline (Call & Text): if you are in a crisis and need help immediately, call 988 or

continue to contact Crisis Text Line by texting “NEDA” to 741741 to be connected with a

trained volunteer at Crisis Text Line. Crisis Text Line is a separate organization staffed by

volunteers who provide free, 24/7 support via text message to individuals who are

struggling with mental health, including eating disorders, and are experiencing crisis

situations.

**Is there anything else you feel we should have asked or that you would like to tell us?**

________________________________________________________________
